# IGH Translocations in Chinese Patients With Chronic Lymphocytic Leukemia: Clinicopathologic Characteristics and Genetic Profile

**DOI:** 10.3389/fonc.2022.858523

**Published:** 2022-06-02

**Authors:** Qinlu Li, Shugang Xing, Heng Zhang, Xiao Mao, Min Xiao, Ying Wang

**Affiliations:** Department of Hematology, Tongji Hospital, Tongji Medical College, Huazhong University of Science and Technology, Wuhan, China

**Keywords:** next-generation sequencing, prognostic biomarkers, cytogenetics, clinical molecular genetics, chronic lymphocytic leukemia, IGH translocation

## Abstract

Immunoglobulin heavy chain translocations (IGH-t) have occasionally been reported in Chinese patients with chronic lymphocytic leukemia (CLL). The objective of the present study was to identify the clinicopathologic features of patients with IGH-t CLL and compare them with those of patients with non-IGH-t CLL. We performed fluorescence *in situ* hybridization (FISH) based on a routine CLL prognostic FISH panel using IGH, IGH-BCL2, *BCL3*, IGH-CMYC, and *BCL6* FISH probes. Furthermore, we retrospectively evaluated the clinical features of 138 newly diagnosed CLL patients *via* chromosome banding analysis (CBA), FISH, and targeted next-generation sequencing. IGH-t was identified in 25 patients (18.1%). Patients with IGH-t CLL had lower flow scores than those with non-IGH-t CLL. The most frequent translocation was t(14;18) (10 patients), followed by t(14;19) (3 patients), and t(2;14)(p13;q32), t(7;14)(q21.2;q12), t(9;14)(p13;q32) (3 patients). The remaining nine patients included three with abnormal karyotypes without translocation involving 14q32, four with a normal karyotype, and two who failed CBA. The most frequently concomitant FISH-detected aberrations were 13q deletion, followed by +12 and *TP53* deletion, while one case involved *ATM* deletion. Complex karyotypes were detected in five patients with IGH-t CLL, in whom all partner genes were non-*BCL2*. Available mutational information indicated that *KMT2D* mutation was the most frequent mutation among tested 70 patients, while *TP53* mutation was the most frequent mutation in the IGH-t group. Moreover, the IGH-t group had higher *FBXW7* (P=0.014) and *ATM* (P=0.004) mutations than the non-IGH-t group, and this difference was statistically significant. Our study demonstrates that IGH-t is not uncommon among Chinese CLL patients, and that its partner genes are multiple. The gene mutational profile of the IGH-t group was distinct from that of the non-IGH-t group, and the concomitant chromosomal abnormalities within the IGH-t CLL group differed. Thus, identification of IGH-t and its partner genes in CLL patients may help further refine risk stratification and strengthen the accurate management in CLL patients.

## Introduction

The advent of ascertainment techniques has led to the revelation that the prevalence of chronic lymphocytic leukemia (CLL), a very common hematological malignancy in Western countries, was on the rise in China (1, 2). The remarkable clinical and genetic heterogeneity of CLL makes prognostic risk stratification vital. Cytogenetic analyses, including conventional banding analysis (CBA) and fluorescence *in situ* hybridization (FISH), play key roles in the differential diagnosis and accurate prognosis of CLL (3).

The use of immunostimulatory CpG-oligonucleotide DSP30 plus interleukin 2 (IL-2), a CLL B cell-specific mitogen in cultures (4), has improved the success rate of CBA, resulting in the discovery of novel chromosomal aberrations in addition to well-known prognostic genetic markers, such as 13q14, 17p13, and 11q22 deletions, and trisomy 12 (+12). Notably, this has enabled recent studies to demonstrate that CLL patients were characterized by a higher incidence of immunoglobulin heavy chain gene (IGH) translocation, a condition which was formerly considered as a rare aberration. Besides, CLL patients showing IGH translocations (IGH-t CLL) were seen as representing a unique subgroup, the prognosis for which was categorized as intermediate–adverse (5). IGH translocation, which results from the juxtaposing of IGH and various partner genes, leading to the activation of a proto-oncogene *via* relocation close to active regulatory sequences, is considered a common cytogenetic marker of B-cell lymphoma and plasma cell myeloma. Therefore, we focused on CLL patients carrying the chromosome 14q32 rearrangement involving IGH, which may cause secondary pathogenesis by deregulating IGH partner genes.

Currently, large cohort studies pertaining to IGH-t CLL are few, and studies investigating this aberration in Chinese CLL patients are rare. Moreover, previous studies have shown that the clinical and molecular features harbored by Chinese CLL patients are different from those of the CLL patients in Western countries (6). In order to clarify the clinicopathological features of IGH-t in Chinese CLL patients, we performed an additional IGH FISH analysis based on the CLL prognosis FISH panel recommended by the 2020 NCCN guidelines (7), and combined it with CBA and the targeted next-generation sequencing (NGS) of a 157-gene panel in 138 CLL patients from our center to identify partner chromosomes, analyze correlations with concomitant cytogenetic aberrations and gene mutations, and compare these characteristics with those of CLL patients without IGH-t abnormalities (non-IGH-t CLL).

## Materials and Methods

### Patients

A total of 138 patients who had been newly diagnosed with CLL, based on the iwCLL criteria (8), were admitted to the Hematology Department of Tongji Hospital from 2019 to 2021. In total, 123 bone marrow and 15 peripheral blood samples were subjected to cytogenetic analyses, including conventional karyotyping and FISH. We also retrospectively analyzed the clinicopathologic features, immunophenotypes, and immunoglobulin heavy variable (IGHV) mutation status. NGS of 70 samples were performed. Samples with positive IGH-CCND1 probe were excluded from the study.

### Morphology and Immunophenotyping Analyses

Using the standard Wright-Giemsa staining protocol, peripheral blood smears were prepared for manual 100-cell differential white blood cell counts. Additionally, manual 200-cell differential white blood cell counts were performed using bone marrow aspirate smears. Particular attention was paid to lymphocytic features.

As previously described (9), the panel of monoclonal antibodies used in our laboratory included antibodies against CD5, CD10, CD11c, CD19, CD20, CD22, CD23, CD25, CD38, CD45, CD79b, FMC-7, CD2, CD3, CD4, CD7, CD8, CD56, and CD10, as well as the immunoglobulin κ and λ light chains (BD Biosciences). We calculated the Matutes score for each case according to the method proposed by Matutes et al. (10). Samples with the score of 4–5 were considered as typical immunophenotypes, while samples with the score of 3 were considered as atypical immunophenotypes. Only samples with a score above 3 were enrolled in our study.

### Conventional Cytogenetics

Cells (10^6^ cells/mL) were cultured with CpG-oligonucleotide DSP30 (2 μmol/L) plus IL-2 (0.2 μg/mL) (11) for 72 h and harvested, following which banding was performed according to standard procedures. Where possible, at least 20 metaphases were analyzed, and description of karyotypes was performed according to the International System for Human Cytogenetic Nomenclature (ISCN 2016). Any abnormal clone with chromosome gains present in at least two different metaphases and chromosome deletions observed in at least three metaphases was defined as a structural aberration. A complex karyotype (CK) was defined as having at least three independent abnormalities (7). CK cases with 3 or 4 aberrations were defined as low-CK or intermediate-CK, respectively. Cases with ≥ 5 aberrations were defined as high-CK (12).

### Fluorescence *In Situ* Hybridization (FISH)

All samples underwent interphase FISH which was performed using a commercial prognostic probe panel (MetaSystems, Altlussheim, Germany), including del(13q14)-*D13S19*, +12-CEP12, del(11q22)-*ATM*, and del(17p13)-*TP53*. In addition to routine panel probes, an IGH break-apart translocation probe was used to identify IGH-t. Based on the presence or absence of IGH-t, the whole patient cohort was divided into two groups: the IGH-t group and the non-IGH-t group. Next, t (13, 14) (q32;q21) IGH-BCL2, t (8, 13) (q24;q32) IGH-CMYC dual color dual fusion translocation probes, and 19q13-*BCL3* and 3q27- *BCL6* break-apart probes were used to identify IGH partner genes in the IGH-t group. Moreover, the t (11, 13) (q13;q12) IGH-CCND1 dual color dual fusion translocation probe was used to rule out mantle cell lymphoma (MCL). Based on the FISH risk classification, described by Rigolin et al. (15), the patients were categorized into three groups: the favorable group (isolated 13q14 deletion or absence of FISH aberrations); the unfavorable group (11q22 or 17p13 deletions); and the intermediate group (+12). The probe cut-off values were as follows: 5% for the deletion probe; 3% for the trisomy probe; 5% for the break-apart probe; and 1% for the dual color dual fusion translocation probe.

### IGHV Mutations and NGS

Genomic DNA was isolated from mononuclear cells from bone marrow or blood samples with the QIAamp DNA Blood Kit (Qiagen, Germany) and used for IGHV mutational status assessment and NGS gene panel analysis.

IGHV gene rearrangements were sequenced by Sanger sequencing using an automated genetic analyser (3500 ABI Applied Biosystems, Foster City, CA, USA). Obtained sequences were analyzed using the International ImMunoGeneTics (IMGT) information system and database tools (IMGT/V-Quest, http://imgt.org). The IGHV mutational status was designated as unmutated, if there were ≤ 2% mutations (> 98% identity), or mutated, if there were > 2% mutations (≤ 98% identity), compared to germline sequences.

As previously described (13), 68 genomic DNA samples and 2 ctDNA (samples 76 and 125) underwent NGS in our laboratory with a panel targeting 157 genes (**Supplementary Table S1**) which are frequently altered in B-cell lymphoma, according to several previously published large-scale cohort studies. Using genome build hg19/GRCh37 as a reference, a sequencing panel covering exons and adjacent 5 intronic base pairs flanking exonic regions in 157 genes was designed online (Designstudio Sequencing, Illumina, San Diego, USA). Sequencing libraries were prepared with AmpliSeq™ Library PLUS for Illumina, using 20 ng of input genomic DNA per sample. Using paired-end 150 sequencing, library sequencing was performed to 2000× coverage on a NextSeq™ 550 system using an Illumina NextSeq™ 500/550 High Output v2 Kit (Illumina, San Diego, USA). Alignment and variant calling were performed using a DNA Amplicon workflow with default parameters on BaseSpace Sequence Hub (Illumina). The generated variants were further annotated using Annovar. Variant filtering was performed by the following steps: 1) select nonsynonymous variants and small insertions/deletions (indels) that overlap coding exons, splice sites; 2) exclude variants with population frequency > 0.0001 in the gnomAD database unless variant is included as a somatic variant of lymphoid neoplasm in the COSMIC database; 3) exclude variants present in an in-house curated blacklist. 4) exclude variants with quality less than 30 or read depth less than 20. For activation-induced cytidine deaminase (AID) somatic hypermutation (SHM) analysis, we additionally selected synonymous variants and variants in intron/UTR regions, and each variant also needed to fulfill the aforementioned criteria from step 2 to step 4.

In addition, some low frequent mutations from case 76 and 125 were listed in red mark in **Supplementary Table S2** because they were validated by the new NGS methods which increased the depth of sequencing to above 10000× by adopting the unique molecular identifiers (UMI) enrichment and target capture DNA library preparation technology. ctDNA from them was extracted from 10ml of plasma by the QIAamp Circulating Nucleic Acid Kit (Qiagen). The NGS library constructed using NadPrep DNA Library Preparation Kit (IDT). Statistical analysis was performed with Fgbio tools (Version 1.2.0).

### Statistical Analysis

Descriptive statistics of quantitative variables included counts and frequency distributions, while whole statistical measurements were based on medians. Chi square tests and Fisher’s exact tests were used to assess associations between categorical variables. Statistical significance was set at P≤ 0.05. Statistical analysis was performed using SPSS 23.0.

## Results

### FISH Results

**Figure 1:**
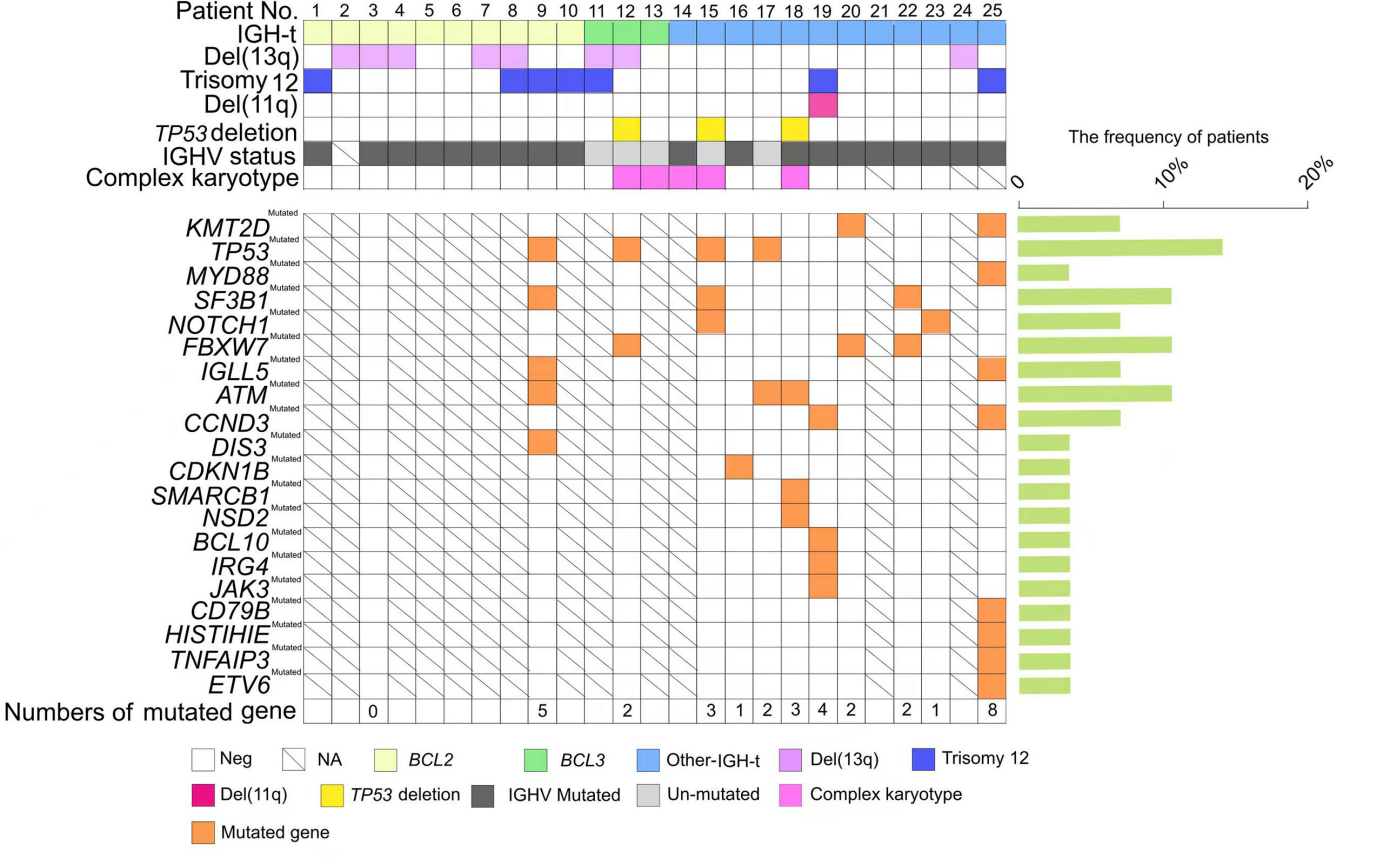
Genetic profiles of chronic lymphocytic leukemia (CLL) patients with immunoglobulin heavy chain (IGH) translocation. Each column represents one patient (n = 25); CLL patients with IGH translocation are clustered into three groups: IGH-BCL2, depicted in light yellow; IGH-BCL3, depicted in light green; and other IGH-t, depicted in light blue. Each row pertains to one genetic factor, which is listed on the left side including del(13q), trisomy 12, del(11q), *TP53* deletion, IGHV status, and complex karyotype. IGHV unmutated status is represented by light grey and IGHV mutated status is represented by dark grey. The presence of a certain mutated gene is shown in orange, and the frequent percentages (%) are listed on the right histogram. Parameters are not available for codes with a slash. Blank code: parameter negative. Colored code: parameter positive.

FISH analysis was performed on samples from all 138 patients enrolled. 98 (71%) patients were positive for at least one of the four probes that were used, as follows: del(13q14)- *D13S19* deletion (28.3%); +12 (21.0%), del(11q22)-*ATM* deletion (10.9%); and del(17p13)-*TP53* deletion (8.7%). Based on the FISH risk classification, 66% of the patients were allocated to the favorable group, 21.0% to the intermediate group, and 19.6% to the unfavorable group (**Table 1**). IGH-t was observed in 25 samples (18.1%), of which 10 (7.2%) carried IGH-BCL2 translocation and 3 (2.2%) carried IGH-BCL3 translocation, and none were positive for *BCL6* or IGH-CMYC (**Figure 1**), as confirmed by FISH. Based on IGH and partner gene FISH results, the IGH-t patients were divided into three groups: IGH-BCL2 group, IGH-BCL3 group, and other IGH-t partner gene group (IGH-others). The remaining 113 patients were assigned to the non-IGH-t group. In the IGH-t group, concomitant FISH aberrations were detected in 15 cases including del(13q) in 8 cases, +12 in 7 cases, del(17p) in 3 cases, and del(11q) in 1 case. Moreover, in the non-IGH-t group, 83 cases had the concomitant aberrations, including the most frequent abnormalities of del(13q) (31 cases), +12 (22 cases), del(11q) (14 cases), and del(17p) (9 cases). Therefore, there was no significant difference between the concomitant FISH abnormalities seen in the IGH-t and non-IGH-t groups (**Table 1**).

**Table 1:** Concomitant FISH results of all patients and those in the IGH-t and non-IGH-t groups.

FISH	All patients (%) n=138	IGH-t (%) n=25	non-IGH-t (%) n=113	P-value
Normal (low risk)	52 (37.7)	10 (40)	40 (35.4)	0.665
13q- (low risk)	39 (28.3)	8 (32)	31 (27.4)	0.646
+12 (intermediate risk)	29 (21)	7 (28)	22 (19.5)	0.343
11q- (high risk)	15 (10.9)	1 (4)	14 (12.4)	0.322
17q- (high risk)	12 (8.7)	3 (12)	9 (8)	0.798

FISH, fluorescence in situ hybridization; Risk stratifications (low, intermediate, high risk) were based on the FISH risk category classification described by Rigolin et al.

### Clinical Features

Baseline clinical characteristics and groupwise comparison for those with and without IGH translocation are shown in **Table 2**. The median age was 61 years (range, 27–84 years). The sex ratio was 2.29:1, with 96 males and 42 females. Patients were divided into two major categories: IGH-t group and non-IGH-t group. The IGH-t group contained three subgroups: IGH-BCL2 group, IGH-BCL3 group, other IGH-t partner gene group (IGH-others) as mentioned above. Of the ten IGH-BCL2-positive cases, all but one had typical morphological characteristics, including small lymphocytes with scant cytoplasm and clumped chromatin. Moreover, all cases with IGH-BCL3 exhibited an atypical CLL morphology characterized by a heterogeneous mixture of small to medium cells, abundant cytoplasm, and indented nuclei, as indicated in a previous study of ours (9). Approximately half the cases with IGH-others displayed atypical CLL morphology. A total of 29 samples had unmutated IGHV status, including 4 in the IGH-t group and 25 in the non-IGH-t group. There was no significant difference between the sex, clinical stage, elevated β2-microglobulin status, or IGHV status readings of the IGH-t and non-IGH-t groups. Furthermore, the flow score (P=0.032) of IGH-t group was lower than that of the non-IGH-t group.

**Table 2:** Baseline characteristics of all patients with CLL and those in the IGH-t (IGH-BCL2, IGH-BCL3, IGH-others) and non-IGH-t groups.

	All patients (n=138)	IGH-t (n=25)			non-IGH-t (n=113)	IGH-t *vs*non-IGH-t (P-value)
		IGH-BCL2 (n=10)	IGH-BCL3(n=3)	IGH-others (n=12)		
Median age (range, years)	61 (27–84)	61 (46–77)	54 (46–56)	62 (40–75)	61 (27–84)	0.619
Male	96	8	3	9	76	0.311
Rai stage						0.198
0–II	64	7	1	7	49	
III–IV	74	3	2	5	64	
Binet stage						0.690
A	43	5	0	3	35	
B	36	3	2	3	28	
C	59	2	1	6	50	
Elevated β_2_ MG	37	2	2	4	29	0.691
Flow score						0.032
3	37	1	2	8	26	
≥4	101	9	1	4	87	
Unmutated-IGHV	29	0	3	2	24	0.362
IGHV-ND	35	1	0	0	34	
CK	22	0	2	3	17	0.540

CLL, Chronic lymphocytic lymphoma; IGH, immunoglobulin heavy chain gene; FISH, fluorescence in situ hybridization; IGHV, immunoglobulin heavy variable; g_2_ MG, β_2_-microglobulin; ND, data not done; CK, complex karyotype.

### Conventional Banding Analysis

Using a CpG stimulant, we successfully analyzed the metaphase karyotypes in 125 (90.6%) samples including 23 in the IGH-t group (**Table 3**) and 102 in the non-IGH-t group. Among the 125 samples, 60 (48%) exhibited an abnormal karyotype involving numerical and structural abnormalities with respect to almost all chromosomes; the abnormalities included translocations, derivative or marker chromosomes, isochromosomes, deletions, insertions, and additions. Among these, balanced and unbalanced chromosome translocations were detected in 24.8% (31/125) of the samples. Overall, the most frequent translocation break point was 14q32, followed by 18q21. Other chromosomal translocations that were identified included three cases of whole arm translocation der (7, 8)(q10;p10), der(10;17)(q10;q10), and der(17;18)(q10;q10); two cases of robertsonian translocation rob(13;21)(q10;q10) and rob(15;22)(q10;q10); and other translocations, including t(1;3)(q32;q27), t(1;12)(q21;p13), t(6;8)(q13;p21), and der (20)t(1;20)(q21;p13). The most frequent numerical aberration detected *via* karyotyping was +12, which was found in 23 samples. Other recurrent aberrations involving chromosome loci hotspots were as follows: duplication of 1q21; deletions of 6q, 11q22, and 13q14; and translocation involving 12p13, and 20p13. Notably, 22 (17.6%) had a complex karyotype (CK) showing more than three chromosomal aberrations. In the IGH-t group, five samples (21.7%) exhibited CKs, including one case of low CK and four of high-CKs. In the non-IGH-t group, seventeen samples (16.7%) exhibited CKs, including five cases of low-CKs, one case of intermediate-CK, and eleven cases of high-CKs. There was no significant difference on aspect of CK in the two groups (P=0.540); (**Table 2**).

**Table 3:** Clinicopathologic features and cytogenetic karyotype results of 25 patients with IGH gene rearrangement.

Case No.	Sex	Age(years)	Rai	Flow Score	Karyotype
1	M	77	0	4	47,XY,+12,t(14;18)(q32;q21)[15]/46,XY[5]
2	M	49	IV	4	46,XY,t(14;18)(q32;q21)[10]
3	M	69	0	4–5	46,XY,del(13)(q14),t(14;18)(q32;q21)[5] /46,XY[15]
4	M	70	I	4	46,XY,t(14;18)(q32;q21)[3]/46,XY[12]
5	M	58	IV	3	46,XY,t(14;18)(q32;q21)[10]
6	F	60	0	4	47,XX, t(14;18)(q32;q21),+18[10]
7	F	65	I	4–5	46,XX,del(13)(q12q22),t(14;18)(q32;q21)[2]/46,XX[18]
8	M	46	I	4–5	47,XY,+12,t(14;18)(q32;q21)[5]/46,XY[5]
9	M	59	III	4	47,XY,+12,t(14;18)(q32;q21)[3]/46,XY[7]
10	M	62	II	4	47,XY,+12,t(14;18)(q32;q21)[13]/46,XY[7]
11	M	63	IV	3	47,XY,+12,t(14;19)(q32;q13)[20]
12	M	54	IV	4	44,XY,t(6;8)(q13;p21),t(14;19)(q32;q13), add(17)(p13),-18,-20[2]/46,XY[18]
13	M	46	I	3	85~90,XXYY,del(6)(q21),-7,-8,-10,+12,-13,t(14;19)(q32;q13)×2,-15,-18[cp10]
14	F	40	II	3	46,XX[9]/46,XX,del(6)(q24)[1]/46,XX,t(2;14)(p13;q32),del(6)(q13),del(11)(q14)[10]
15	M	73	I	3	45,X,-Y,del(3)(p24),der(4)t(4;11)(p16;q14),del(6)(q23),del(7)(q32),t(7;14)(q21.2;q12),del(17)(p13),add(19)(q13.4)[10]
16	M	50	IV	3	46,XY,t(9;14)(p13;q32)[5]/46,XY[5]
17	M	56	IV	4	46,XY,i(8)(q10),add(18)(p11)[10]
18	M	65	III	3	46,XY,+1,del(1)(p22),t(1;21)(p34;p11),del(4)(q28),add(4)(q35),add(5)(p15), add(6)(p24),add(7)(p22),t(7;13)(p21;q21),add(12)(q24),add(17)(p13), add(17)(q25),del(18)(p11)add(19)(p13),add(21)(q22),+20,+21,+22[cp10]
19	F	72	0	3	47,XX,dup(1)(q12q44),+12[7]/46,XX[13]
20	M	50	I	4–5	46,XY[13]
21	M	55	I	4	46,XY[20]
22	M	75	II	3	46,XY[20]
23	M	56	IV	4–5	46,XY[20]
24	F	65	II	3	NA
25	M	61	IV	3	NA

F, female; M, male; NA, not available; ND, not done; FISH, fluorescence in situ hybridization; IGHV, immunoglobulin heavy variable.

### Next-Generation Sequencing

NGS of 70 samples was conducted. Of these, 44 (62.9%) carried at least one mutation and 30 (42.9%) had ≥2 mutations in the targeted NGS region. Detailed lists of mutation detected in 70 samples are shown in **Supplementary Table S2**. The most frequent mutations listed involved the genes *KMT2D* (15.7%), *TP53* (12.9%), *SF3B1* (8.6%), *IGLL5* (8.6%), *NOTCH1* (7.1%), *MYD88* (7.1%), and *FBXW7* (5.7%). 12 of the 25 IGH-t samples underwent NGS, and 11 (91.7%) showed positive results. The most frequent mutations in the IGH-t group involved *TP53*, *KMT2D*, *NOTCH1*, and *IGLL5.* Other relatively rare mutations were detected in the genes, *CCND3* (2 cases), *MYD88* (1 case), *HIST1H1E* (1 case), and *CD79B* (1 case). In total, 58 of the 113 non-IGH-t samples underwent NGS, and 33 (56.9%) were positive and the most frequent mutations in this group were found in *KMT2D* and *TP53.* However, no significant differences were found between the two genes in the IGH-t and non-IGH-t groups (P=0.921; P=0.064, respectively). The IGH-t group showed higher *FBXW7* (P=0.014) and *ATM* (P=0.004) mutations than the non-IGH-t group, and the difference was statistically significant (**Table 4**). Mutational details are shown in **Figure 1**.

**Table 4:** Mutation results of all patients and those in the IGH-t and non-IGH-t groups.

Mutation	All patients (%) n=70	IGH-t (%) n=12	non-IGH-t (%) n=58	P-value
*KMT2D*	11 (15.7)	2 (16.7)	9 (15.5)	0.921
*TP53*	9 (12.9)	4 (33.3)	5 (8.6)	0.064
*IGLL5*	6 (8.6)	2 (16.7)	4 (6.9)	0.593
*MYD88*	5 (7.1)	1 (8.3)	4 (7)	0.999
*NOTCH1*	5 (7.1)	2 (16.7)	3 (5.1)	0.201
*FBXW7*	4 (5.7)	3 (25)	1 (1.7)	0.014
*ATM*	3 (4.3)	3 (25)	0 (0)	0.004

## Discussion

IGH translocation reportedly induces malignancy *via* the placement of a strong transcriptional enhancer from the IGH locus near a proto-oncogene normally located on the partner chromosome, thereby overriding normal gene transcriptional regulation (16). The findings of our study indicated that chromosome 14q32-IGH translocation occurred in 18.1% of the patients, with an incidence rate slightly higher than the range of 3.1–15% indicated by independent studies conducted in Western countries (14, 17, 18), but similar to that (17.9–19.6%) reported by other Chinese groups (19, 20). The difference observed between the IGH-t and non-IGH-t CLL groups, suggested that due consideration should be given to IGH-t screening in patients with low flow scores. The most frequently concomitant aberrations detected by FISH were 13q deletions, followed by +12 and *TP53* deletions, while only one case involving an *ATM* deletion was observed. No difference was found between the concomitant FISH abnormalities found in the IGH-t and non-IGH-t groups.

*BCL2* was the most common partner gene found in the 25 patients with IGH-t, while all 10 patients positive for IGH-BCL2 showed t(14;18)(q32;q21) in CBA, with none showing CK. As previously reported by us, another three IGH-BCL3-positive patients showed t(14;19)(q32;p11) in CBA, with two showing a CK (9). Although translocation partner genes in the remaining 12 patients were not identified by FISH due to the limited number of partner gene probes used, CBA did reveal the partner chromosomes of t(2;14)(p13;q32), t(7;14)(q21.2;q12), and t(9;14)(p13;q32) in patients 14, 15, and 16, respectively. Furthermore, patients 14 and 15 showed a CK, and based on a combination of previous reports and our findings, we speculated that the partner genes *BCL11A*, *CDK6*, and *PAX5* were involved in these 2 cases (21). Notably, although there were seven patients with IGH-t, CBA showed normal (four cases) or abnormal karyotypes regardless of IGH-t (three cases). We speculated that CBA may have missed IGH translocations due to several reasons: (i) some cryptic translocations with IGH, such as t(4;14), t(14;16), which are common in multiple myeloma could not be detected by CBA due to the translocated chromosomal fragments being too small to be visible; (ii) subtle chromosomal changes caused by atypical or complex chromosomal changes, including insertions, inversions, and multiple chromosomal translocations, may escape CBA detection and thus may have to be further verified *via* DNA NGS; (iii) hematopoietic cells with normal karyotypes were analyzed and it was found that leukemic cells were in the nondividing phase. Among three cases showing abnormal karyotypes regardless of IGH-t, case 18 had CK in CBA, and the remaining two patients (cases 17 and 19) already carried two chromosomal abnormalities combined with cryptic IGH translocation, which could be considered as a CK. Thus, the IGH probe further refined the prognostic stratification of these two patients. To our knowledge, a CK, defined as the presence of at least three chromosomal abnormalities in the same clone, is emerging as a new negative prognostic biomarker of an adverse outcome and worse response to chemoimmunotherapy and novel agents (12). There was no difference between the IGH-t and non-IGH-t groups with respect to the incidence of CK. However, partner genes of the five IGH-t patients included *BCL3* or IGH-others, but not *BCL2*, as well as four cases who were high-CK. Therefore, based on a previous study (17) which indicates that patients with IGH-BCL3 would receive poorer prognoses compared to those with IGH-BCL2, we considered whether the presence of a CK was one of the causes leading to poor prognoses in the IGH-BCL3 and IGH-others groups.

Presently, studies investigating gene mutations in patients with IGH-t CLL are rare. However, a few studies have proposed that Asian CLL patients may be characterized by an inherent landscape of gene mutations of their own that are distinct from those observed in Western CLL patients (22, 23). Our mutational analysis revealed that *KMT2D* was the most frequent mutation in CLL patients, which was substantiated by another study by Yi et al., which indicated that the frequency of mutations observed in *MYD88* and *KMT2D* of Chinese CLL patients was higher compared to that observed with Western CLL patients (24). In addition, patients with IGH-t CLL experienced a higher incidence of mutations than patients with non-IGH-t CLL. Furthermore, *TP53* was the gene that was found to undergo mutations most frequently in patients with IGH-t CLL, a finding that was not consistent with that of the largest IGH-t CLL study conducted by Pérez-Carretero et al., which indicated that the most frequently mutated genes were *NOTCH1* and *IGLL5* (25). There may be a few possible reasons for this discrepancy. First, some studies have confirmed that Chinese CLL patients are different from Western CLL patients, in terms of the type and frequency of gene mutations. Second, the sample size used to obtain gene mutation data was small, and the distribution of partner genes in our study mainly comprised *BCL3* or IGH*-*others, which differed from the results of the study by Pérez-Carretero et al. Results from another study showed that patients with IGH-BCL2 CLL had a favorable prognosis (14, 26), similar to that observed in the Dohner hierarchical FISH low-risk group. Previous studies conducted by us, and other groups have indicated that patients with IGH-BCL3 CLL usually experience an aggressive clinical course with atypical features (9, 18). Interestingly, the frequencies of *FBXW7* and *ATM* mutations detected in patients with IGH-t CLL were much higher than those in patients with non-IGH-t CLL. Therefore, our findings further clarified that Chinese CLL patients displayed distinct phenotypic and genotypic features. The group of IGH-t CLL patients was heterogeneous with different genetic profiles and pathogenic characteristics. Thus, they should be further subdivided according to different partner genes in order to allow for more accurate prognostic evaluations.

This retrospective study was beset with certain limitations. First, the number of cases in this study was small, especially with insufficient NGS data from the group of patients with IGH-BCL2, limiting the power of the statistical analysis. Second, the therapeutic implications for patients in different groups were not evaluated.

In conclusion, to the best of our knowledge, the current study is the first to analyze Chinese IGH-t CLL patients using a combination of CBA, FISH, and genetic mutation analyses. Our study revealed that IGH-t is not an uncommon event in Chinese CLL patients, and that t(14;18)-*BCL2* is the most frequent partner gene. Chinese CLL patients exhibit unique mutation characteristics compared to Western CLL patients. Additionally, genetic mutations varied between the IGH-t and non-IGH-t groups. Moreover, concomitant karyotype abnormalities and gene mutational profiles were different within the IGH-t CLL group due to different partner genes. Therefore, it is important to routinely identify this category of CLL patients, using IGH probes, and to subdivide them depending on the partner genes involved in the translocations, which can be detected *via* a combination of CBA, FISH, and NGS. Thus, a larger study which further clarifies the heterogeneity of IGH-t CLL in order to help strengthen the prognostic stratification of CLLs and choose the reasonable therapeutic strategy in the era of targeted treatment may be warranted.

## Data Availability Statement

The datasets presented in this study can be found in online repositories. The names of the repository/repositories and accession number(s) can be found below: https://www.ncbi.nlm.nih.gov/sra; PRJNA669583.

## Ethics Statement

The studies involving human participants were reviewed and approved by the Ethics Committee of Tongji Hospital of Tongji Medical College of Huazhong University of Science and Technology. The patients/participants provided their written informed consent to participate in this study. Written informed consent was obtained from the individual(s) for the publication of any potentially identifiable images or data included in this article.

## Author Contributions

QL collected clinical sample and related data, designed the study and wrote the manuscript. YW reviewed all related literature, performed bioinformatic analysis, and revised the manuscript in this study. SX performed FISH experiment and gathered related data. HZ made chromosome banding analysis. XM performed the flow cytometry test. MX performed high throughput sequencing experiment. All authors contributed to the article and approved the submitted version.

## Conflict of Interest

The authors declare that the research was conducted in the absence of any commercial or financial relationships that could be construed as a potential conflict of interest.

## Publisher’s Note

All claims expressed in this article are solely those of the authors and do not necessarily represent those of their affiliated organizations, or those of the publisher, the editors and the reviewers. Any product that may be evaluated in this article, or claim that may be made by its manufacturer, is not guaranteed or endorsed by the publisher.
