# Supplementary material for: IGH Translocations in Chinese Patients With Chronic Lymphocytic Leukemia: Clinicopathologic Characteristics and Genetic Profile
Source: Front Oncol. 2022 Jun 2;12:858523. doi: 10.3389/fonc.2022.858523 (PMC9201519; doi:10.3389/fonc.2022.858523)
Supplement: Supplementary file 1 [file Table_1.docx]

Supplementary Table S1.

Gene list of 157 targeted next-generation sequencing study

ABL1, ACTG1, AKT1, ARID1A, ATM, ATP6AP1, ATP6V1B2, B2M, BCL10, BCL11B, BCL2, BCL6, BCOR, BIRC3, BRAF, BTG1, BTG2, BTK, CARD11, CASP10, CCND1, CCND3, CCR4, CCR7, CD28, CD58, CD70, CD79A, CD79B, CD83, CDKN1B, CDKN2A, CHD2, CNOT3, CREBBP, CRLF2, CTNNB1, CXCR4, DDX3X, DIS3, DNM2, DNMT3A, DTX1, DUSP2, EBF1, EED, EGR1, EGR2, EIF2A, EP300, ETV6, EZH2, FAM46C, FAS, FAT1, FBXW7, FGFR3, FLT3, FOXO1, FYN, GATA3, GNA13, GNAQ, GPR183, HIF1A, HIST1H1B, HIST1H1C, HIST1H1D, HIST1H1E, HNRNPA2B1, HRAS, HVCN1, ID3, IDH1, IDH2, IGLL5, IKBKB, IKZF1, IKZF3, IL7R, IRF4, ITPKB, JAK1, JAK2, JAK3, KDM6A, KIT, KLF2, KLHL6, KMT2C, KMT2D, KRAS, LTB, MAP2K1, MAP3K14, MAPK1, MAX, MED12, MEF2B, MYC, MYD88, NF1, NFE2, NFKBIE, NOTCH1, NOTCH2, NRAS, NT5C2, PAX5, PHF6, PIK3CA, PIK3R1, PIM1, PLCG1, PLCG2, POT1, POU2AF1, POU2F2, PRDM1, PRKCB, PTEN, PTPN1, PTPN11, RB1, RHOA, RPL10, RPS15, RRAGC, SAMHD1, SETD2, SF3B1, SGK1, SH2B3, SMARCA4, SMARCB1, SOCS1, STAT3, STAT5B, STAT6, TBL1XR1, TCF3, TET1, TET2, TMSB4X, TNFAIP3, TNFRSF14, TNFRSF1B, TP53, TRAF3, TRRAP, U2AF1, USP7, VAV1, VMA21, WHSC1, WT1, XPO1
